# The cell rejuvenation atlas: leveraging network biology to identify master regulators of rejuvenation strategies

**DOI:** 10.18632/aging.206105

**Published:** 2024-09-09

**Authors:** Javier Arcos Hodar, Sascha Jung, Mohamed Soudy, Sybille Barvaux, Antonio del Sol

**Affiliations:** 1 Computational Biology Group, CIC bioGUNE-BRTA (Basque Research and Technology Alliance), Bizkaia Technology Park, Derio, Spain; 2 Computational Biology Group, Luxembourg Centre for Systems Biomedicine (LCSB), University of Luxembourg, Esch-sur-Alzette L-4362, Luxembourg; 3 Biomedical Data Science Group, Luxembourg Centre for Systems Biomedicine (LCSB), University of Luxembourg, Esch-sur-Alzette L-4362, Luxembourg; 4 Ikerbasque, Basque Foundation for Science, Bilbao, Bizkaia 48012, Spain

**Keywords:** rejuvenation, computational biology, scRNA-seq, aging, database, cellular biology

## Abstract

Current rejuvenation strategies, which range from calorie restriction to *in vivo* partial reprogramming, only improve a few specific cellular processes. In addition, the molecular mechanisms underlying these approaches are largely unknown, which hinders the design of more holistic cellular rejuvenation strategies. To address this issue, we developed SINGULAR (Single-cell RNA-seq Investigation of Rejuvenation Agents and Longevity), a cell rejuvenation atlas that provides a unified system biology analysis of diverse rejuvenation strategies across multiple organs at single-cell resolution. In particular, we leverage network biology approaches to characterize and compare the effects of each strategy at the level of intracellular signaling, cell-cell communication, and transcriptional regulation. As a result, we identified master regulators orchestrating the rejuvenation response and propose that targeting a combination of them leads to a more holistic improvement of age-dysregulated cellular processes. Thus, the interactive database accompanying SINGULAR is expected to facilitate the future design of synthetic rejuvenation interventions.

## INTRODUCTION

There is a growing interest in rejuvenation interventions for their potential to mitigate the effects of aging in humans. These interventions, ranging from lifestyle changes such as calorie restriction and exercising over gene therapies like partial reprogramming up to surgical procedures as heterochronic parabiosis, have been shown to improve various biological aging markers [1] and to increase the average lifespan in several model organisms [2–5]. However, they suffer from two main limitations. On one hand, although these interventions proved efficacious in improving specific cellular processes, none of them achieves a holistic functional improvement across tissues. In this regard, a review of pharmacological approaches to slow aging identified mostly specific and non-overlapping effects on different hallmarks of aging [6]. On the other hand, clinical translation of current rejuvenation strategies is often not feasible (parabiosis), bears significant safety concerns (partial reprogramming) or requires sustained lifestyle changes that are known to have low compliance (calorie restriction, exercise). In order to mitigate these issues, it is imperative to characterize and compare current interventions at different levels of biological organization to enable the discovery of more comprehensive rejuvenation strategies that correct a wider array of dysregulated biological processes.

Elucidating commonalities and differences of the effects of diverse rejuvenation strategies on different cell types and cellular processes remains a challenge. Although a wealth of high-resolution transcriptomic data has been produced, every study employs different quality control metrics and downstream processing pipelines, which impedes a direct comparison of the obtained insights. For instance, the parameters for filtering low quality cells are largely inconsistent in three major atlases of calorie restriction [7], heterochronic parabiosis [8] and exercise [9]. Moreover, the depth of the analysis varies from study to study. While some studies focus on describing the transcriptional changes caused by an intervention alone [10], others include computational modeling approaches to interrogate effects on the gene regulatory network or cell-cell interactome [7–8]. Nevertheless, the use of different computational tools largely prohibits a direct comparison even though the same kind of analyses were performed in different studies. Thus, in order to rigorously compare the effects of rejuvenation strategies, it is crucial to unify the processing of the data and the subsequent analysis.

Computational network biology approaches have shown great success in providing mechanistic insights by linking different scales of biological organization, including transcriptional regulation, intracellular signaling and intercellular communication, thereby generating testable hypotheses [11]. For instance, pre-existing work has explored the changes in transcription factor (TF) activity associated with age by using network approaches to estimate TF expression based on the presence of its regulons [12]. As such, these approaches would allow the characterization of the rejuvenation effects on different cellular processes determined by signaling and transcriptional regulation as well as cell-cell communication. Moreover, following a network-based approach allows the identification of master regulators of each strategy and combining them could enable a more holistic rejuvenation, i.e., a more complete set of rejuvenated cellular processes.

Here, we introduce SINGULAR (Single-cell RNA-Seq Investigation of Rejuvenation Agents and Longevity), a cell rejuvenation atlas that characterizes the response to cellular rejuvenation strategies at the single-cell level in a unified analysis framework. In particular, we propose to view aging as a metastable transcriptional state associated with loss of regular physiological function. Conversely, rejuvenation entails the conversion from an aged to a more youthful transcriptional state.

In this regard, we characterized the effect of 6 rejuvenation strategies across 9 studies on 73 cell types at the gene regulatory network, intracellular signaling, cell-cell communication and cellular process level. Moreover, we identified master regulators at every level of biological organization and identified common targets across immune cells. Finally, we exemplify how SINGULAR can be exploited to select drugs that could mimic the effect of complex interventions. Thus, we expect SINGULAR to be of great utility in informing further advances in human age reversal.

## RESULTS

### A unified processing and analysis pipeline for single-cell based rejuvenation studies

To overcome the abiding issue of heterogeneous processing and analysis approaches between different studies, we propose a unified multiscale analysis pipeline that allows for the characterization and comparison of the effects of rejuvenation interventions. Starting from quantified expression profiles of single-cell RNA-seq experiments from treated and untreated donor samples, our pipeline first filters low quality cells based on dynamic thresholds for the percentage of mitochondrial and ribosomal reads as well as the relationship of read counts to detected genes. Next, the expression profiles of all cells in a dataset after regressing out the effect of cell cycle induced differences and normalization using scTransform [13]. Finally, the optimal clustering of cells is automatically identified by maximizing the Calinski-Harabasz Index [14].

After processing the data, our pipeline analyzes each dataset of treated and untreated samples at different levels of biological organization. Initially, it characterizes differentially expressed genes and the cellular processes they belong to. Subsequently, transcriptional regulatory networks (TRN) among differentially expressed genes by following a previously published method [15]. In brief, assuming an “inhibition dominant” regulatory logic in which one upregulated inhibitor is sufficient to cause the downregulation of a gene (no matter the number of activating relationships), a prior knowledge network (pkn) of TF - gene interactions is pruned to remove interactions in which the gene activity is behaving differently to what is seen in the differential expression profile. As a next step, our pipeline integrates signaling and transcriptional regulation to reconstruct sustained signaling cascades and identify their key molecules using SigHotSpotter [16]. Eventually, we employ InterCom to interrogate intercellular communication by reconstructing cell-cell interactions mediated by ligands and their cognate receptors [17]. In brief, InterCom infers ligand-receptor interactions by modeling intracellular signaling and downstream TF expression to ensure compatibility.

**Figure 1:**
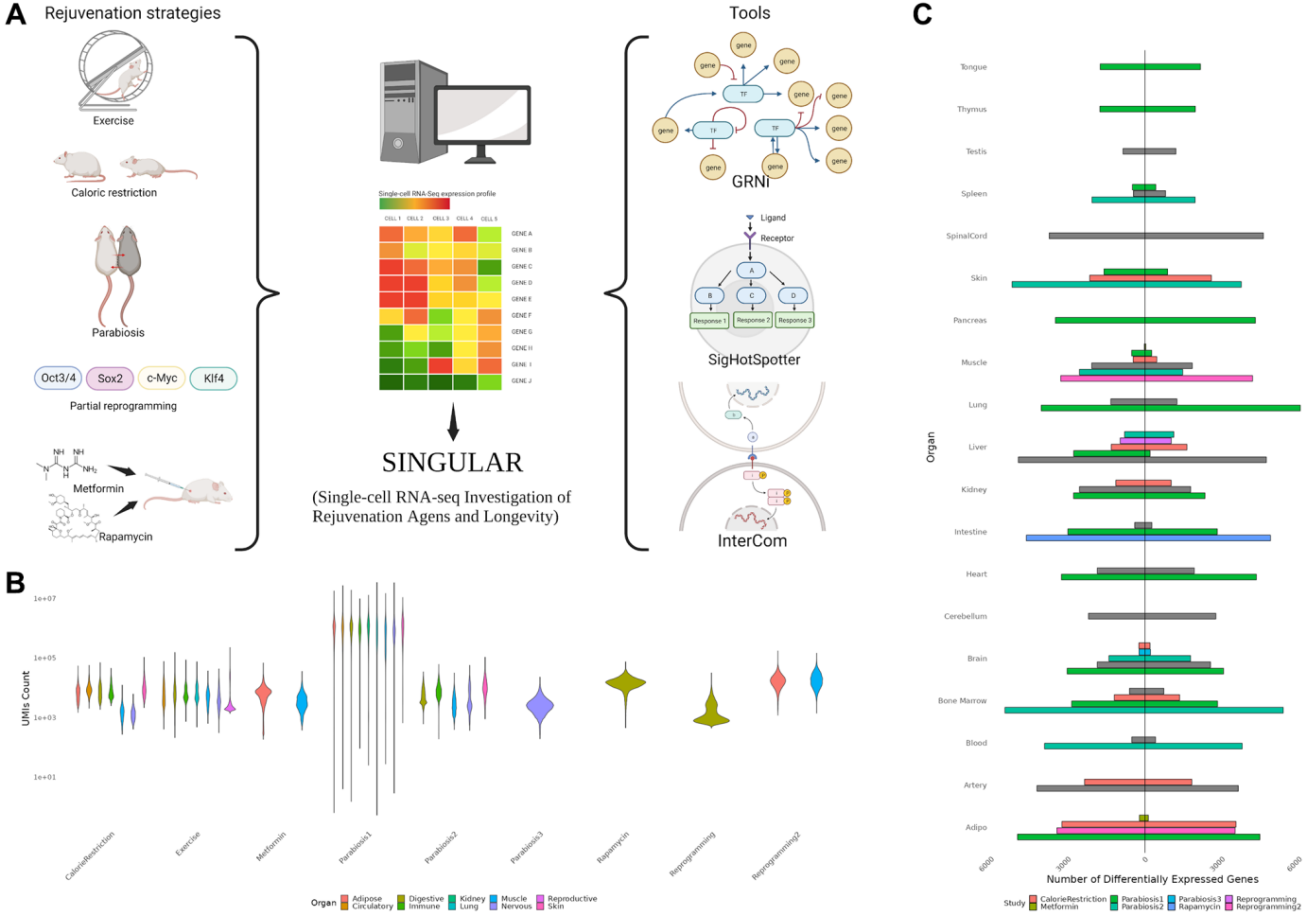
(**A**) Overview of the SINGULAR project and its initial motivation. Publicly available datasets for several rejuvenation interventions were analyzed in this study. With the exception of Parabiosis, analyzed from three datasets, Reprogramming, which was analyzed from two datasets, and Metformin, also used in the Rapamycin experiment condition, every condition had data from one study. SINGULAR combines a unified processing pipeline for all the datasets with three main tools to explore transcriptional regulatory networks, signaling pathways, and cell-to-cell ligand receptor interactions. (**B**) Example comparison of the technical heterogeneity motivating this study. UMI counts across organ systems and studies, as well as the organs sample in each of the different studies, both vary greatly. (**C**) Comparison of counts of unique upregulated and downregulated genes from different studies grouped at the organ level, derived using the Delegate method. Even after applying SINGULAR’s unified preprocessing pipeline, substantial heterogeneity by organ and study in the transcriptional response to rejuvenation remains.

We collected 9 previously published single-cell RNA-seq datasets of heterochronic parabiosis (3 datasets), calorie restriction (1), exercise (1), metformin (2), rapamycin (1) and *in vivo* partial reprogramming (2) [7–10, 18–22] (Figure 1A, Supplementary Table 1, Supplementary Figure 1). As expected, we observed substantial technical variability in these datasets evidenced by large differences in sequencing depth, which further underscores the need for a homogeneous data processing pipeline (Figure 1B). Altogether, the employed datasets span a total of 74 cell types across 18 organs. Notably, tissues from the central nervous system, adipose tissue, liver and bone marrow could be found in multiple intervention datasets. To begin with, we set out to characterize the differential expression patterns of each cell type in each organ in response to the individual rejuvenation strategies and identified a considerable heterogeneity (Figure 1C). While systemic interventions such as calorie restriction and heterochronic parabiosis consistently exert large effects on the transcriptome of multiple organs, metformin has little to no effect on the organs it has been examined in. Interestingly, although exercising is directly affecting the muscles by diverting blood to them, the largest transcriptional effects were observed in the liver, artery and spinal cord.

### Identification of transcriptional master regulators that mediate rejuvenation effects

**Figure 2:**
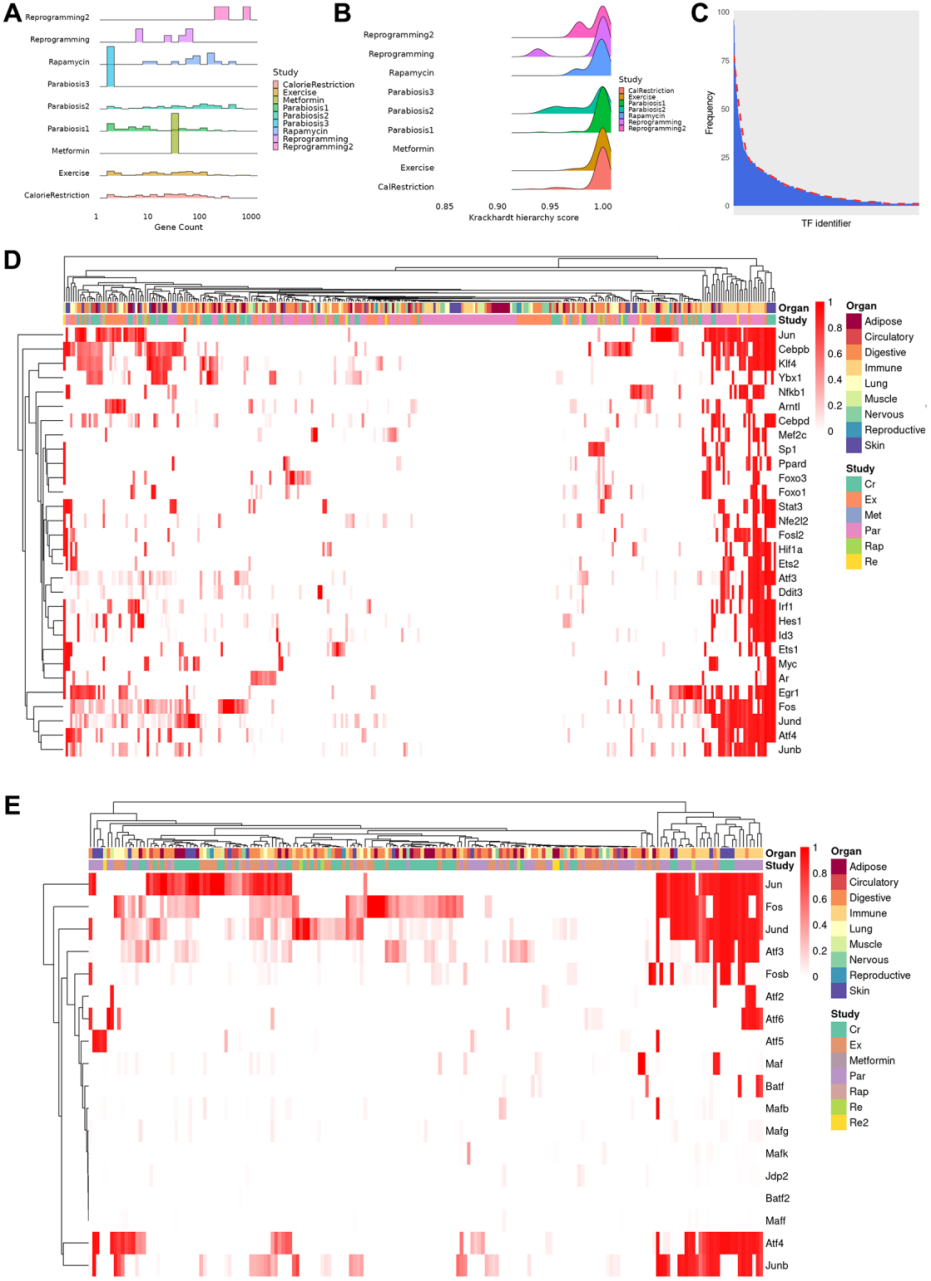
**Properties and clustering of master regulators in the rejuvenation response.** (**A**) Ridge plot of network size, calculated as sum of unique TFs and targets for each regulatory gene network, grouped by study (bin size = 30, average number of genes 72, median number of genes 31, range 2–867). Provided enough distinct regulatory networks are observed, their number of elements can vary between organs and cell types of the same dataset. (**B**) Krackhardt hierarchy scores of all TRNs. In this case, we universally see values very close to 1 (mean 0.994, rage 0.932–1), indicating a very hierarchical regulatory response for all rejuvenation interventions. This motivated the search for master regulators in the transcriptional networks. (**C**) Distribution of instances of a specific TF being observed in each of the TRNs. The majority of TFs are seen in only a few regulatory networks, but a minority appear in a significant fraction. (**D**) Heatmap of the TF score (see online methods) for the 30 TFs with the greatest average ranking across all TRNs. Clustering was performed with the manhattan distance and the McQuitty method. Coordinated TF responses can be observed, as well as activity patterns strongly associated more with the rejuvenation condition than the cell type, potentially uncovering more holistic rejuvenation interventions by targeting master regulators behind different interventions. (**E**) Heatmap subset transcription factors known to be part of the AP-1 complex. Several clusters that contain a single master regulator can be observed in the differential rejuvenation response. Given these cofactors are expected to be coexpressed, this suggests a rejuvenation response in immune cell types under Calorie Restriction and Parabiosis that relies on the action of distinct AP-1 dimers.

In order to gain insights into the regulatory relationships explaining the observed differential expression profile, we reconstructed TRNs among differentially expressed genes for each cell type in different tissues. As a result, we obtained 317 TRNs of cell types that were affected by a rejuvenation intervention. On average, TRNs are composed of 72 genes (range: 2–867) although most networks contain less than 35 genes (Figure 2A). Interestingly, the size of the TRNs is only weakly related to the number of differentially expressed genes (Pearson correlation, r = 0.21, *p* < 0.001), which suggests that the transcriptional response to rejuvenation interventions is dependent on other regulatory mechanisms. In this regard, we hypothesized that signaling dependent TFs, whose activity is not only mediated by their expression level but also extracellular signals, may regulate the genes that cannot be explained in the TRNs. Indeed, using a previously curated collection of signal-dependent TFs [23], we found between 77.8% and 100% (median 87.97%) of genes that are differentially expressed, have a known potential regulator but are not part of the TRNs can be regulated by signaling dependent TFs. In addition, we assessed how hierarchical each network is using the Krackhardt Hierarchy Score and found the TRNs to be highly hierarchical (average: 0.994, range: 0.932–1) (Figure 2B). This indicates the presence of a few ‘master regulators’, i.e., TFs that explain a large fraction of gene expression changes (see Methods for details).

Based on network statistics, we sought to identify these master regulators and simulated the downstream effects of activating a single TF in the network to assess the number of genes whose differential expression could be determined by this gene alone. Thus, a TF with a score of ‘1’ determines all genes in a network while a TF with a score of ‘0’ determines no other gene. Following this approach, we detected 493 TFs with a non-zero score across all cell types, organs and interventions. However, the majority of these TFs only act as master regulators in less than 5 conditions (Figure 2C). Moreover, the master regulator score of many TFs is low across the majority cell types and interventions. For example, the rejuvenation response in adipocytes after exercising is orchestrated by the co-expression of *Clock* and *Arntl*, which induce different downstream factors depending on the organ of origin. On the other hand, *Nfkb* and *Esr1* regulates varying fractions of differentially expressed genes depending on the intervention (Supplementary Figure 2A). Indeed, it is not uncommon that in different conditions both shared and distinct mechanisms are found, suggesting therapeutic approaches to be equally promising through similar mechanisms. In basal cells of the Skin, for instance, *Srf*, *Cebpb*, *Atf4*, *Jun* and *Myc* shared the majority of their downstream regulatory target genes whereas other TFs mostly acted in a non-overlapping manner (Supplementary Figure 2B). Similar patterns could also be found in different cell types of the same intervention, with *Ddit3*, *Spib* and *Cebpb* mediating the effect in the granulocyte lineage while the remainder of the transcription factor response is determined by the maturity of the cell (Supplementary Figure 2C). Not surprisingly, we also observed distinct mediators of the intervention response. For instance, *Ybx1*, *Klf4* and *Ets1* were found to be master regulators of exercise and calorie restriction in hepatocytes, whereas only *Foxo3* attained a high master regulator score in case of heterochronic parabiosis (Supplementary Figure 2D).

Next, we aimed at interrogating the most common intervention mediators and selected the 30 TFs that have the highest average master regulator score across all cell types (Figure 2D). Surprisingly, when contrasted against previously existing analysis that documented substantial declines or increases in TF activity with ageing [12], the overlap with those TFs is limited; with only 4 of our 30 mater regulators appearing in such an analysis (Nfkb1, Irf1, Arntl and Id3). Moreover, the sign of the change in TF activity varied depending on cell type, rather than being consistently positive or negative. This would suggest a marked distinction between the regulatory agents associated with age and those able to orchestrate the rejuvenation response.

Interestingly, our master regulator TFs have been previously associated with diverse cellular functions, including differentiation, proliferation, immune response and cell migration. Intriguingly, when grouping these TFs by their master regulator score in every cell type, we observed the presence of several clusters. As a general observation, we conclude that our master regulators rather group by intervention instead of cell type. In light of the diverse set of enriched cellular processes, this suggests the induction of distinct signaling pathways that differentially activate broad TFs. However, there exists one cluster that almost exclusively contains immune cells after treatment of heterochronic parabiosis or calorie restriction (Figure 2D; right part). Although all of these TFs contribute to the mediation of the intervention effects, *Jun*, *Junb*, *Jund*, *Atf4* and *Fos*, all of which belong to the AP-1 transcription factor complex, display a consistent involvement (Figure 2E). In addition, we observed that many smaller clusters are formed that predominantly contain a single master regulator even though the AP-1 complex TFs mostly co-occur. The presence of the AP-1 complex across multiple interventions prompted us to dissect the involvement of all known subunits [24] separately. Consistent with the known dimerization patterns of the AP-1 complex [25], *Fos* and *Jun* clearly emerged as the most common master regulators across interventions and cell types. In contrast, other *Jun*-, *Fos*- and *Atf*-family TFs act more selectively as master regulatory, which leads us to hypothesize that the cell type and intervention specific effects are exerted by distinct AP-1 dimers. Interestingly, although previous studies have documented the influence of this complex in promoting age-related inflammation (“inflammaging”) [26], our analysis strongly suggests their action as anti-aging mediators depending on their dimerization.

To support the involvement of the identified master regulator TFs, we cross-referenced the 30 TFs having the highest master regulator score with aging-associated genes contained in GenAge [27]. As a result, we found 53% (16/30) master regulators to be linked to aging with varying degrees of evidence. In particular, *Arntl*, *Cebpb*, *Foxo1* and *Jun* possess strong evidence and have been directly linked to aging in mammalian and non-mammalian model organisms. In addition, the gene products of *Myc* and *Nfe2l2* have been directly linked to aging in a cellular model system and *Foxo3* has been shown to be involved in human longevity. Interestingly, several genes, i.e., *Ar*, *Egr1*, *Jun* and *Sp1*, have been shown to regulate genes previously linked to aging. Less evidence is provided for *Hif1a* and *Nfkb1*, which are known to be involved in pathways or mechanisms linked to aging. Finally, *Ddit3*, *Fos* and *Stat3* are known effectors of aging-related genes.

Despite the TFs that have been found in GenAge, we collected publicly available transcriptomic perturbation data of the top 30 master regulators and applied MultiTIMER [28], a multi-tissue transcriptional aging clock, to quantify potentially rejuvenating effects. Due to the nature of MultiTIMER as a predictor of transcriptional age in bulk data, we chose to validate the master regulators found in SINGULAR with experiments available in the Gene Expression Omnibus (GEO). In particular, we found knockdown/knockout experiments for *Klf4*, *Irf1*, *Atf4*, *Myc*, *Hif1a* and *Esr1* in cell types where they have been identified as master regulators (Supplementary Table 3). Since master regulators are up-regulated upon rejuvenating interventions, we would consequently expect the age of normal cells to increase after their knockdown or knockout. Indeed, we observed increases in the predicted cellular age after perturbing *Klf4* (9.1 years), *Irf1* (3.9 years), *Hif1a* (2.6 years). In addition, we also collected transcriptional profiles after overexpression of *Klf4* and *Myc*. Interestingly, the predicted cellular age after *Myc* overexpression is considerably younger (−9.7 years) whereas it slightly increased in case of *Klf4* (1.6 years). These results suggest that master regulators act synergistically with other TFs to exert a rejuvenating effect in a cell type dependent manner. This idea is further supported by current partial reprogramming strategies that upregulate *Klf4* and *Myc* in combination with *Pou5f1* and *Sox2* to achieve a significantly higher reduction in cellular age compared to what we observed for *Klf4* or *Myc* alone [29].

### Crosstalk between transcriptional master regulators and intracellular signaling response

Most of the TFs with the highest master regulator potential across interventions are well known to be activated or inhibited by multiple signaling pathways. Thus, we set out to identify the active signaling molecules that are likely to mediate the activation of master regulators in each cell type, tissue and intervention, as described before. Moreover, we compared the accordingly detected signaling molecules in treated and untreated samples to select those that are differentially active between both conditions. As a result, we identified 452 molecules in 33 cell types across conditions and organs. Of these molecules, 74 directly activated the downstream TFs of the corresponding TRNs after treatments. The full set of results can be viewed in the SINGULAR database.

**Figure 3:**
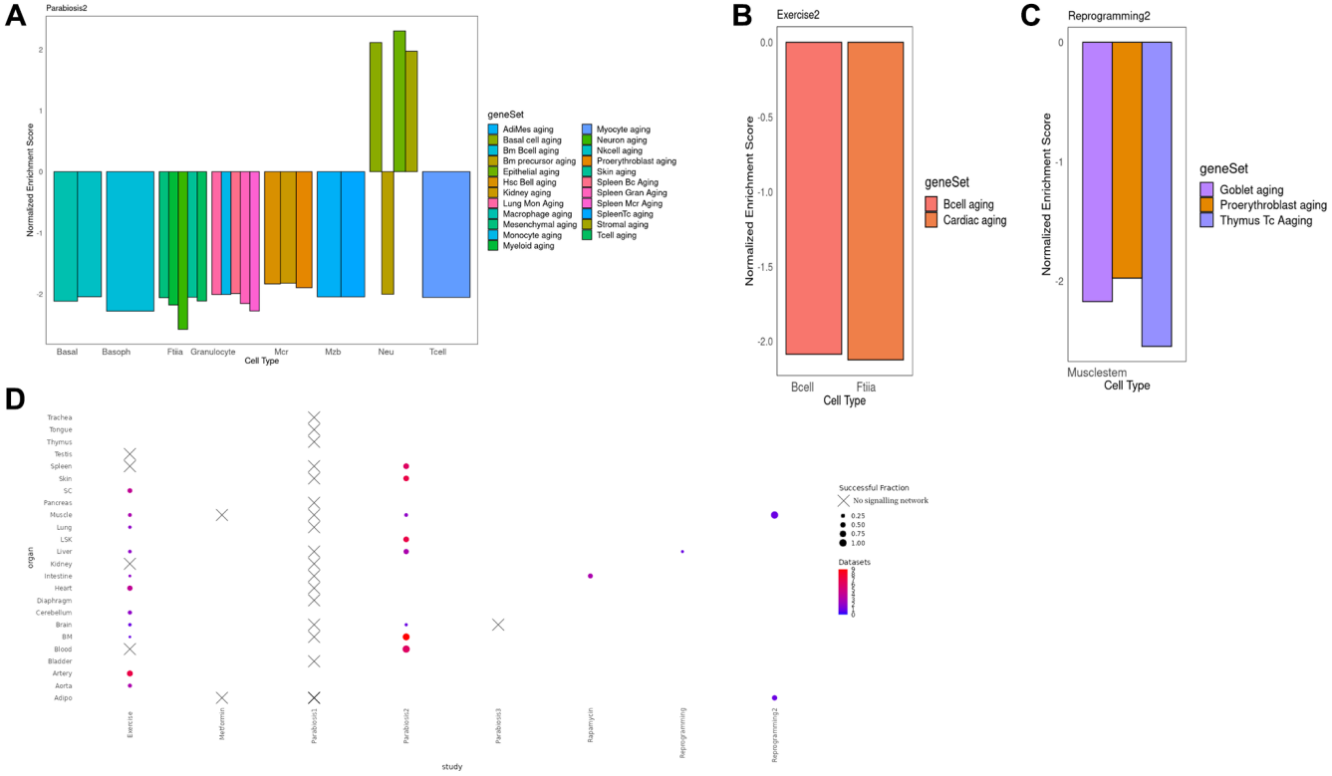
(**A**) Normalized enrichment scores for different cell types in different organs in the Parabiosis2 dataset. We observe substantial heterogeneity, including situations where a cell is negatively enriched both for its actual cell type and for the aging signature of other, very different lineages. This suggests that Parabiosis may be the most comprehensive rejuvenation intervention at this level of analysis. It must be noted that Neutrophils were the only cell type with a mixed rejuvenating and aging signature, but this is consistent with known responses to heterochronic parabiosis experiments. (**B**) Normalized enrichment score for the shared component between the TRN and the signaling network for Muscle fiber and Lung B cells under the Exercise condition. In this example, the geneSet cell markers for an aged transcriptome perfectly match the celltype the multi-modal network was derived from. (**C**) Normalized enrichment score for the shared component between the TRN and the signaling network in Reprogramming dataset for Muscle stem cells. In this example, the negative aging signature is found for three different cell types, none matching the one the data was derived from. (**D**) Bubble plot illustrating the number of cell types per organ and the fraction of cell types per organ where we were able to detect a sustained signalling network associated with the rejuvenation condition, per organ and study. Crosses indicate absence for any cell type. Full equivalence between the geneSet legend labels and the Tabula Muris Senis enrichments can be found in Supplementary Table 2.

To interrogate the function of the integrated signaling cascades and their induced TRNs, we performed Gene Set Enrichment Analysis (GSEA) of their constituent genes [30]. Strikingly, 23 integrated networks are negatively enriched in aging gene sets derived from Tabula Muris Senis, as found in The Molecular Signatures Database (MSigDB) [31]. This finding supports the rejuvenating effects of different interventions on cell types on the basis of an independent dataset (Figure 3A–3C). After heterochronic parabiosis however, neutrophils predominantly displayed a pro-aging signature (Figure 3D). This is consistent with a previous study reporting an increase of a gene signature of neutrophil activation in hematopoietic stem and progenitor cells after parabiosis treatment [8]. Of note, especially in the case of parabiosis, we observed a reduction of aging signatures derived from various tissues. Nevertheless, in other cases, the enriched signatures precisely matched the tested cell type, such as in case of lung B cells where Grb2 mediates the activation of the transcriptional master regulator Fos (Figure 3B, Supplementary Figure 3A).

Despite the reversal of pro-aging signatures in multiple cell types, we observed other enriched functions that are consistent with our current understanding of the interventions. For instance, E2f target genes, cell cycle control and the G2m checkpoint were negatively enriched after partial reprogramming of the muscle, which displays an expected decrease in cellular proliferation that is consistent with previous studies indicating an up-regulation of the cell cycle inhibitor Cdkn1a in the critical treatment window [32] (Supplementary Figure 3B). Moreover, in the same experiment, cardiac muscle organogenesis was positively enriched suggesting an ongoing re-commitment to a fully differentiated state after de-differentiation (Figure 3C, Supplementary Figure 3B). Interestingly, while Neutrophils in the bone marrow show signs of overall rejuvenation in response to heterochronic parabiosis, their inflammatory potential increases (Supplementary Figure 3C). This implies that parabiosis could have harmful effect on aging alongside its rejuvenating benefits. Finally, the response of T cells in the peripheral blood to Parabiosis2 was mediated by the kinase Pak2 and the master regulators Nfkb1 and Stat3. As a result, we observed a decline in the Pi3k/Akt/mTOR signaling pathway, which is a well-known aging determinant. In fact, hypomorphic Pi3k mice show an increased longevity (Supplementary Figure 3D).

### Integration of gene network inference, signaling and intercellular communication analysis

Based on our finding that the transcriptional response to several interventions could be linked to sustained signaling cascades that get activated, we finally aimed at interrogating whether these effects are induced by ligand-receptor mediated cell-cell interactions. Thus, we employed InterCom [17] to reconstruct the cell-cell communication networks of each treated and untreated organ. Similar to our assessment of the intracellular signaling cascades, we focus in the remainder on the most significant interactions that are unique to the treated condition and that involve a receptor as a key signaling molecule. The complete information can be accessed in the SINGULAR database.

**Figure 4:**
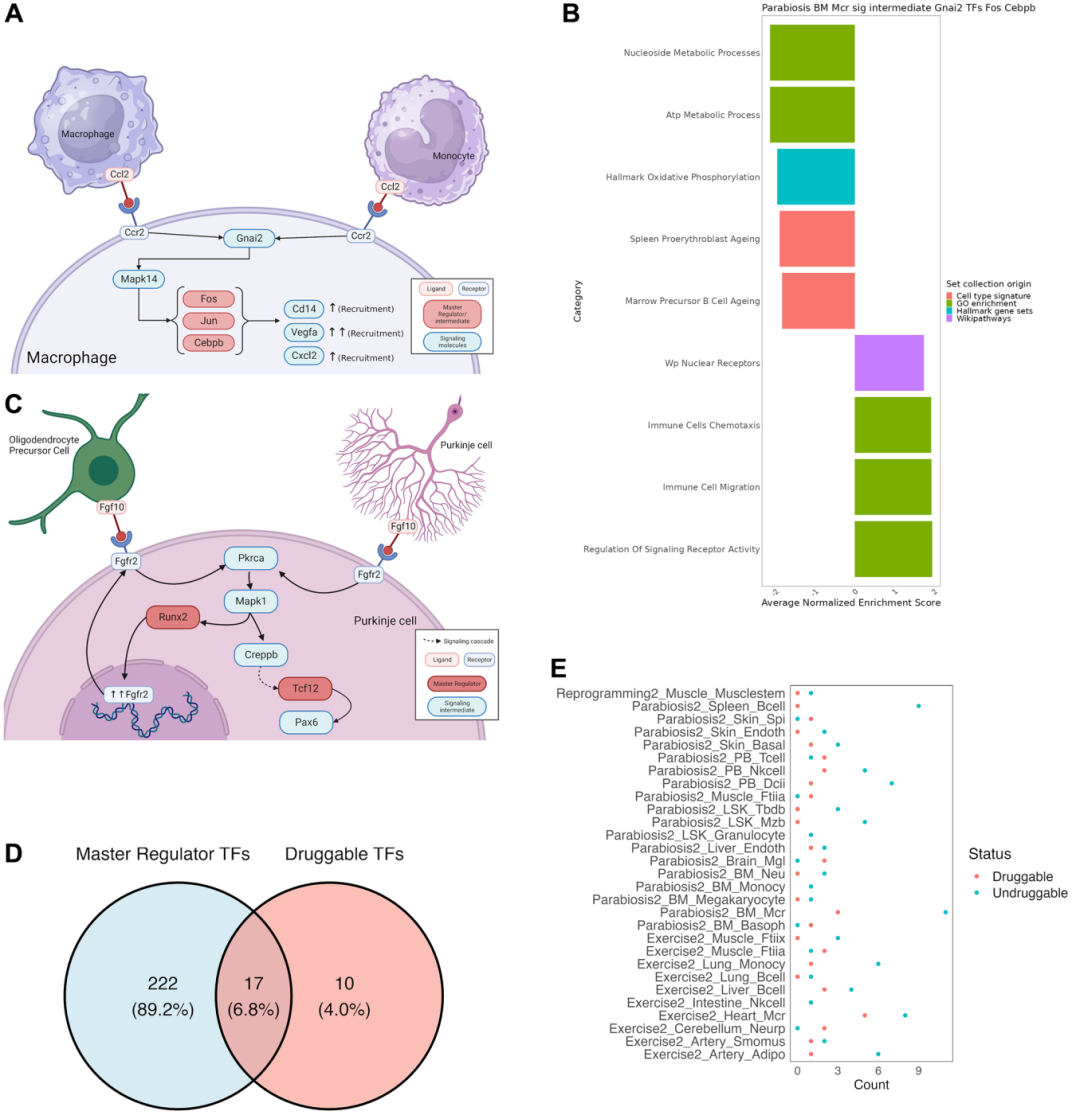
**Mechanistic insights from a combined approach of all tools used by SINGULAR.** (**A**) Recapitulation of well documented cell communication pathway for Macrophage recruitment under the Parabiosis condition. Ccr2 recognizes Ccl2, which initiates a signaling cascade to activate the AP-1 complex, which leads to the activation of chemotaxis genes. (**B**) Further validation of this well-known pathway from gene set Enrichment analysis of the members of the connected component of TRN and signaling cascade crosstalk for Gnai2 as a signalling intermediate and Fos, Jun and Cepbp as TRN TFs. Values have been averaged from several related functions, full results in Supplementary Table 4. (**C**) Novel signaling cascade. In Purkinje cells of the cerebellum, Fgf10 binds to Fgfr2, initiating a cascade in which Pkrca leads to the activation of Mapk1, which recruits Runx2 for further expression of the Fgfr2 receptor, as well as a separate Creppb-mediated signaling cascade that ends with Tcf12 activating Pax6, a transcription factor known for its neuroprotective properties. (**D**) Intersection between druggable activating TFs in DrugBank and the master regulators uncovered in this study. (**E**) Number of druggable key signaling molecules for every integrated TRN and signalling cascade.

Deriving mechanistic insights and testable hypotheses from the response to different rejuvenation strategies could significantly accelerate the development of new anti-aging treatments. Therefore, we focus on two illustrative examples to demonstrate the potentiality of our unified analysis approach. First, we found our analysis to recapitulate known cell communication effects in macrophages after heterochronic parabiosis (Figure 4A). More specifically, our analysis revealed that Gnai2 activates the AP-1 complex genes Jun and Fos upon dissociation of Ccr2 upon recognition of its cognate ligand Ccl2 [33]. Activation of the AP-1 complex in turn leads to the up-regulation of known chemotaxis related genes, such as Cd14, Cxcl2 and Vegfa [34–36]. Indeed, positive enrichment of chemotaxis-related gene sets in a GSEA of the signaling cascades and downstream TFs underscored the chemotactic expression program induced by Ccr2 (Figure 4B). As a second example we chose to illustrate a novel, non-canonical signaling cascade that has not been reported before. In response to exercising, Purkinje cells in the cerebellum form an autocrine loop and interact with oligodendrocyte precursor cells via the Fgf10-Fgfr2 axis (Figure 4C). While our analysis recapitulates the downstream activation of Runx2, which in turn up-regulates and guarantees the expression of Fgfr2, we found that Pax6 is activated by Tcf12 in response to Fgfr2 activation. Although the function of Pax6 has not been reported in Purkinje cells, it is a known neuroprotective transcription factor [37].

### Identification of potential drugs targeting key TFs and signaling molecules

In order to demonstrate the utility of SINGULAR, we asked whether we can determine drugs that can target the identified TF master regulators and key signaling molecules. For that, we collected all available drug-target relationships in DrugBank and searched for drugs that could activate our master regulators or mimic the effect of rejuvenation interventions on key signaling molecules. For this purpose, we classified TFs as master regulators if they determine at least 30% of the network TFs when activated according to our simulation studies (see online Methods). Unsurprisingly, of the 239 transcriptional master regulators across all cell types, organs and interventions, only 17 could be activated by drugs (Figure 4D). Moreover, these TFs predominantly belong to the class of nuclear receptors, including *Nr3c1*, *Vdr*, *Nr1i2*, *Rxra* and *Ar*. However, notable exceptions are the AP-1 complex proteins *Jun* and *Fos* as well *Trp53*. These further underscores the suitability to interfere with the AP-1 complex to mimic the effect of complex interventions. In order to determine whether any of these drugs possess known rejuvenating effects, we cross-referenced them with DrugAge, a database of aging related drugs [38]. As a result, we found several compounds with demonstrated effects on lifespan in model organisms. For instance, Curcumin, a Vdr agonist, extends the maximum lifespan of *D. melanogaster* on average by 19.5% at high concentrations and Vitamin D3 extends the average lifespan of *C. elegans* by 26.8% in a dose-dependent manner. Moreover, Bezafibrate, a partial agonist of Nr1i2, has been shown to increase the average lifespan of *C. elegans* by 13%. In contrast to TFs, the differentially active key signaling molecules between treated and untreated conditions are generally better druggable (Figure 4E). In particular, the microglia specific key signaling molecules after parabiosis *App* and *Mapk14* are targeted by 24 and 56 drugs, respectively. However, none of the identified molecules target both genes.

## DISCUSSION

In this study, we performed a unified analysis of different rejuvenation interventions, with the goal of leveraging network biology to provide a rigorous comparison of their effects and mediators at different scales of biological organization. In doing so, we uncovered several master regulators orchestrating the rejuvenation response, and compared their influence across different organs, experiments and cell types.

Our approach successfully identified several previously known age-related TFs. For instance, we found Arntl to be a master regulator in rejuvenation, corroborating its earlier identification as the TF with the most significant age-related decline in activity in at least one prior analysis [12]. However, only three other matching TFs were identified, with the sign of TF activity changes varying substantially by cell type. This suggests notable differences between transcriptional changes associated with aging and the regulators of rejuvenation. It also uncovered previously undocumented mediators of rejuvenation interventions. Moreover, in cases where the transcriptional mediators are known, our analysis provides novel insights. For example, while the AP-1 complex formed by Fos and Jun has been described to regulate diverse cell functions, and in particular the inflammaging response, our analysis further demonstrates that different subunits and cofactors serve as master regulators of the response to specific interventions. In light of our findings and a recent study that highlighted an up-regulation of the Jun-Fos dimer expression, which is accompanied by increasing inflammation, it is plausible that AP-1 dimers composed of other subunits are responsible for inducing anti-aging effects [24]. Indeed, although AP-1 binds to a palindromic DNA motif, its specificity is conveyed by the bZIP subunits [39]. However, directing the dimerization as a potential therapeutic approach remains a challenge due to the heterogeneous involvement of subunits in different cell types. Moreover, transcription factors have long been considered “undruggable” and only incremental progress has been made, which aggravates the search for potential interventions that could be translated to the clinics [40]. Apart from the AP-1 complex, our analysis revealed the transcriptional stress response TFs NFE2L2 and MAF as master regulators of certain rejuvenation interventions in different cell types. Indeed, MAF and NFE2L2 have been shown to dimerize and regulate gene expression programs that protect against oxidative stress, which are lost with age [41]. Moreover, over-expressing MAF has been shown to rescue these protective expression programs and preserve fitness in an animal aging model [41]. Conversely, the reduced activity of NFE2L2 leads to increased cellular senescence and inflammation [42].

The application of current intervention strategies with the largest effects across tissues, i.e., heterochronic parabiosis and exercising, is impractical in humans. In contrast, our multiscale analysis pipeline sheds light on the regulatory mediators of their effects. This offers the unique opportunity to design new approaches that mimic or combine the effect of these complex interventions in the future. For instance, we showed that immune and skin cell types emerge as having common master regulators across interventions, which suggests them to be amenable targets for intervention. Intriguingly, these immune cells whose rejuvenation is hypothesized to lead to a significantly increased healthspan [43]. As of today, immune system rejuvenation is mostly considered for individual cell types [43]. However, despite stark phenotypic differences of immune cell types, our analysis suggests that rejuvenation to the extent it is achieved in heterochronic parabiosis is possible by targeting a common set of regulators. In addition to mimicking the effects of complex interventions, our analysis also offers the potential to experimentally validate non-overlapping master regulators from different inferred gene regulatory networks for additive or even synergistic benefits. For instance, Ybx1, Klf4, Ets1 and Fos orchestrated the response of hepatocytes to exercising while in the case of parabiosis, Foxo3 appeared to be the sole master regulator. Due to the differences in the transcriptional response, the combined targeting of these TFs is expected to have synergistic effects.

Despite the advantages of our unified analysis pipeline and the utility of SINGULAR we discussed before, our study has a few limitations. First, the comparisons of cell types across interventions suffers from potential biases due to the number of cells gathered for each cell type in each study. Although we did not observe any implications in the datasets we employed for this study, an empirical analysis of the employed tools suggests that small populations of cells (less than 50) typically result in a higher number of false positive interactions. Second, when interrogating the cell-cell communication network, we rely on ligand-receptor mediated interactions. It is well known that other communication channels, such as extracellular vesicles, contribute to the exchange of information between cells. However, incorporating this information requires targeted experimental assays, which are currently still not widely applied. Third, differential expression testing in sc-RNAseq data is always at risk of false negatives due to drop-outs [44], a concern particularly acute with genes with a generally low level of expression like TFs. We nevertheless take steps to mitigate the impact of this concern. For instance, we selected the DELegate method for differential gene expression tests (see Methods) and focused on TFs that consistently appear across organs, cell-types and interventions. Finally, caution is warranted when interpreting the results due to inherent differences in the coverage of organs. For instance, while the effect of metformin was assessed in the intestine, adipose tissue and muscle, only the intestine was profiled in case of rapamycin.

Nevertheless, we believe that SINGULAR is of great utility for better understanding the mechanisms underlying different rejuvenation interventions and to identify novel rejuvenation agents by providing a comprehensive array of target genes in the pursuit of a holistic anti-aging strategy.

## METHODS

### Unified processing of the sc-RNAseq rejuvenation datasets

Data analysis was done in Seurat version 4.3.0 (R versions 4.2.3 and 4.3.0), following a unified, biologically informed approach in preprocessing similar to Subramanian et al. [45].

All datasets were processed from cellranger matrix, feature and barcodes when possible. The main exceptions were the second Parabiosis dataset [8], which was processed from FASTQ files with cellranger 6.1 with all argument set to default except an explicit call to –include-introns to get processed cellranger files, and the second Reprogramming dataset [18], in which the raw count matrix after quality control offered by the authors was used for all downstream analyses, due to the unavailability of the VectorBuilder sequences necessary to build the raw count matrices from the raw reads.

Every dataset was further processed with a unified preprocessing pipeline in which the median absolute deviation argument of the our filterCells function was set to 3 to filter outlying cells based on mitochondrial counts, ribosomal counts, number of features and number of counts, with the latter two further filtered based in a linear model with the formula = log10(nFeature_RNA) ~ log10(nCount_RNA)) for unified quality control.

Resulting Seurat objects were further normalized with the SCT transform function with the vars.to.regress argument with “C.C. difference” for cell cycle adjustment and vst.flavor set to “v2”. Doublets were removed using DoubletFinder version 2.0.3. Cell cycle scoring for adjustment relied on the cell marker database annotation retrieved from Ensembl and provided in the repository as a supplementary file.

### Integration of within-experiment sc-RNA seq datasets and removal of unwanted sources of variation

Integration of the datasets was performed with SCTtransform [13] splitting every Seurat object by biological replicates of the same condition and sex (if any) sex (if only this was available) and condition in every case. rPCA dimensionality reduction was used together with the SCT normalization method.

### Clustering of sc-RNAseq data and cell type identification

LSK and Skin in the second parabiosis dataset [8] were downsampled (keeping original files 1–4 for the former and 5–8 for the later) due to the full data causing extreme numbers of clusters with the Monocle3 clustering function.

Every other dataset was clustered in full after preprocessing with the cluster_cells argument of the Monocle3 library version 3.1, with 10 neighbors, leiden clustering, UMAP used for dimensionality reduction and iterating 5 over several orders of magnitude of the resolution parameter. Every iteration was scored with the Calinski-Harabasz Index and the clustering with the highest score kept, except if more than 30 clusters were detected after this optimizing process, in which case the partitions function of Monocle3 was used instead.

Preliminary cell type assignments were done using SCINA 1.2.0, but this was substantially supplemented by manual annotation with the support of the literature, the cell marker database and the panglao database of single-cell gene markers, as well as support from the markers provided by the original authors of each study, if it was provided.

### Differential gene expression test for sc-RNAseq data

Differential gene expression analysis was in every case performed using the DElegate R package version 1.10 (https://github.com/cancerbits/DElegate) a wrapper to use the DESeq-2 differential gene expression analysis assigning cells to pseudo-replicates. This choice stems from its positive benchmarking against other methods in the metrics of precision, sensitivity and false discovery rate [46]. Moreover, random assignment of pseudo-replicates for pseudo-bulk analyses is the best option to mitigate the concern of drop-outs that is always present in scRNA-seq data. Final results were filtered to only keep those cases when the average log fold change in gene expression had an absolute value greater than 0.25 or the gene was seen in more than 10% of the cells in that cell type for both conditions. When more than one control condition was provided (such as isochronic parabiosis and old untreated) both were taken as the comparison group against the rejuvenation intervention. Comparisons were always made against the old condition, dropping the young data, if any, from further analysis, even if they were used during the integration.

### Gene set enrichment analysis

Analysis of enriched gene ontology functions and KEGG pathways was realized with package WebGestaltR version 0.4.4 and the ORA (overrepresentation analysis) method. Differentially expressed genes at the *p*-value adjust < 0.05 significance level were used as a query, while all the genes in the original count matrix (i.e., the RNA assay Seurat object rownames) were used as background. As illustrated in the attached repository, the remaining arguments were overrepresentation analysis, false discovery rate corrected with the Benjamini-Hochberg with a threshold of 0.05.

### Strategy to identify cell-type specific transcriptional master regulators

Inference of the master regulators uncovered in this study was done using R library GRNOpt, developed for a previous manuscript [15]. Differential gene expression results were booleanized by setting the log fold changes in the rejuvenation condition filtered as described above to 1 if they were positive and to 0 if they were negative. The inhibition dominance logic rule was used for building the transcriptional regulator networks with the prune gurobi function. This method has been utilized in prior studies to investigate cell state transitions, especially in the context of stem cell differentiation. Indeed, this approach is specifically designed to identify master regulators that orchestrate transcriptional shifts responsible for initiating and maintaining changes over time [47].

Every TF in a gene regulatory network was scored by exploring the consequences of initiating the state of every element in the network to undetermined and examining the consequences of activating each TF with a depth-first search algorithm. The fraction of the final state of the network (as described by the booleanized gene expression) that matched all the downstream changes due to this activation was then the TF score, implementing the same inhibition dominance logic rule that is used during the creation of gene regulatory network, in which any number of inhibitory relationships takes precedence (i.e., sets the state to ‘inhibited’) over any number of activating relationships. In this way, a TF in a completely hierarchical connected relationship to the rest of the network that could explain its entire state by being activated would get a score of 1.0, while sparse networks in which not all the elements are connected will always get a fractional score, an end node that was only scored based on predicting its own state would get a score of 1/(N), where N is the size of the network, and an inhibited gene in the booleanized gene expression would get a score of 0, being unable to explain its own state relative to the differential gene expression if activated.

The TF-regulon interactions database from CollecTRI [48] was used as a previous knowledge network. This previous knowledge network was retrieved using the omnipath python library with the omnipath.constants.InteractionDataset. COLLECTRI function with the 10090 argument (to get the interactions collected for mouse). Conversion of Protein IDs to gene symbols was performed with the getBM argument of biomart, with rows with non-matching NA values and those where the consensus stimulation and consensus inhibition entries were not opposites filtered out. Finally, for the Calorie Restriction study (performed on *Rattus norvegicus*) a further conversion of the mice previous knowledge network developed as described above was performed, from the mouse to the rat orthologs, using the “https://dec2021.archive.ensembl.org/” Esembl mirror during the call to the getLDS function.

Endpoint previous knowledge networks for mice and rat are provided in the repository.

Finally, during the development of the gene regulatory network, duplicate interactions and values in the TF “from” column that were not transcription factors were filtered out, both for the previous knowledge network and the booleanized differential gene expression input.

### Discovery of stable signaling networks and signaling hotspots from sc-RNAseq data

For the cell communication results, we used Sighostpotter (https://gitlab.com/srikanth.ravichandran/sigHotSpotter) with a custom modification in the for_plotting_networks_functions.R function .trimResults. Lines 56 and 61 were replaced with res_trimmed <- res_trimmed(res_trimmed(,2)>0.7,) and res_trimmed <- res_trimmed(res_trimmed(,2)<0.3,) respectively, to facilitate downstream analysis. Everything else was as seen in the repository and the modified version is provided with the main repository for a custom install.

sigHotSpotter was selected for its ability to model both canonical and non-canonical signaling networks, which generate locally stable configurations. This modeling approach is ideal for studying the sustained, long-term changes involved in both aging and rejuvenation interventions. Moreover, sigHotSpotter identifies ‘hotspots’ in the cascade that sustain the new cell state.

Results were analyzed with the sigHotSpotter pipeline function with cutoff value set to 30, percentile set to 70, and the RNA assay counts as input matrix.

Signaling networks were kept after following two conditions. First, both the rejuvenation intervention and the control condition needed to have a non-NA results. Then, only signaling networks where the signaling intermediate was activated in one condition and inhibited in another (activation defined as a final score above 0.70, and inhibition as a value below 0.30, NA entries included) and present in the rejuvenation intervention were kept for downstream analysis.

### Cross-talk between TRNs and signalling networks

To identify the cross talk between the master regulator transcriptional networks and the Sighostpotter results, filtered SigHotSpotter results were subset by signaling intermediate and the networks in which the same transcription factor shared a node with outgoing relationships both in the TRN and the SigHotSpotter edges object from column were concatenated to compute the connected components using igraph’s components function with the mode “weak” argument. If there was a single component, the elements of the entire shared network were taken, while if there was more than one, only the connected component with the signaling intermediate was used for downstream analysis.

The elements of this combined network were then queried for Gene Set Enrichment Analysis with the WebGestaltR library (version 0.4.4) using the online enrich method GSEA and three further queries from the Molecular Signatures Database retrieved at https://www.gsea-msigdb.org/gsea/msigdb. In particular, the M2 curated gene sets, M8 cell type signature gene sets and M5 ontology gene sets were used.

### Cell to cell communication analysis in sc-RNA seq data

Intercom was used to model the communication between cells as it pertains to those events that are only seen in treated rejuvenation conditions. For a complete description of how the tool works, we refer to the original work that introduced Intercom [17] but we offer a summarized version as follows:

As a preliminary step, InterCom generates a scaffold of experimentally validated receptor-ligand cell-cell interactions, later integrated with intracellular signaling networks and gene regulatory interactions. For ligand-receptor interactions, a previously curated database [49] had the extent of its relationship narrowed down to only those ligands annotated as ‘Secreted’ in Uniprot.

For the intracellular signaling network scaffold, interactions from databases both publicly available (Omnipath, Reactome) and with limited access (MetaCore from Thomson Reuters) were collected, choosing those related to signal transmission (phosphorylation and ubiquitination events). For transcriptional regulator interactions, we again used Metacore, keeping only direct interactions that were known to entail activation or inhibition. These three elements compose the scaffold.

Regarding the analysis of the provided data, InterCom calculates an interaction score for each potential cell–cell interaction by multiplying the average receptor expression and average ligand expression in all cells of a population expressing the receptor or ligand, respectively.

The significance of these scores is then assessed by comparing the scores of all potential cell–cell interactions contained in the scaffold between the two interacting cell types. Interactions with scores in the top decile are considered significant and are the focus of our analysis.

As it pertains to specific parameters in our analysis, Intercom analysis was performed taking the SCT integration counts as an input matrix and with the sigcutoff and z.score.cutoff parameters set to 0. Every other argument was left as default.

Results were further filtered to keep only the interactions unique to the rejuvenation intervention and with a significance score above 0.90, to keep the top decile as usual, as described above.

### Assessing the effect of MR perturbation on cellular age

To validate the discovered master regulators, we carried out a search of perturbation data the Gene Expression Omnibus (GEO), using ‘knockdown’, ‘knock-out', ‘shRNA’ ‘overexpression’ and ‘knock-in’ as keywords and selecting as ‘Study Type’ both ‘high throughput sequencing’ and ‘expression profiling by array. In addition, in order to keep comparisons informative, we selected datasets that meet the following criteria: (1) The relevant master regulator is perturbed. (2) This master regulator is the only gene perturbed in the experiment. (3) The study does not involve cancer, embryonic, or similarly altered cell lines that would confound the transcriptional age estimation. (4) The master regulator is found in our single-cell data at least once in a closely similar cell type to the one used in the bulk experiment.

All datasets that met these selection criteria and included both control and perturbed data were used as input for MultiTIMER [28], a transcriptional age clock able to generate predictions for any tissue. Identifiers as well as the difference in predicted age can be seen in Supplementary Table 3. Master regulators shown in Figure 2D that are not listed in Supplementary Table 3 either lacked perturbation data or did not meet the specified criteria described above.

Expression data were converted into log2 of the rank of the gene expression and further subset to the intersection of genes present in all datasets before comparing the difference in predicted biological age between experimental data and controls.

### Data availability

The following publicly available datasets were used in the analysis of this study: GEO Accession numbers GSE137869 (Calorie Restriction), GSE176206 and GSE144600 (Reprogramming), GSE193093 and GSE222510 (Parabiosis) as well as GSA CRA004660 (Parabiosis) and CRA007207 (Exercise).

Code for the processing pipeline and auxiliary functions in the workflow is available at https://github.com/jarcoshodar/singularsource.

SINGULAR is available as a publicly available interactive database at https://singular.lcsb.uni.lu/. Source code for a local install and exploration of the data is available at https://git-r3lab.uni.lu/mohamed.soudy/singular.

## Supplementary Materials

Supplementary Figures

Supplementary Tables
