# Supplementary material for: The cell rejuvenation atlas: leveraging network biology to identify master regulators of rejuvenation strategies
Source: Aging (Albany NY). 2024 Sep 9;16(17):12168–90. doi: 10.18632/aging.206105 (PMC11424581; doi:10.18632/aging.206105)
Supplement: Supplementary Figures [file aging-16-206105-s001.pdf]

SUPPLEMENTARY FIGURES

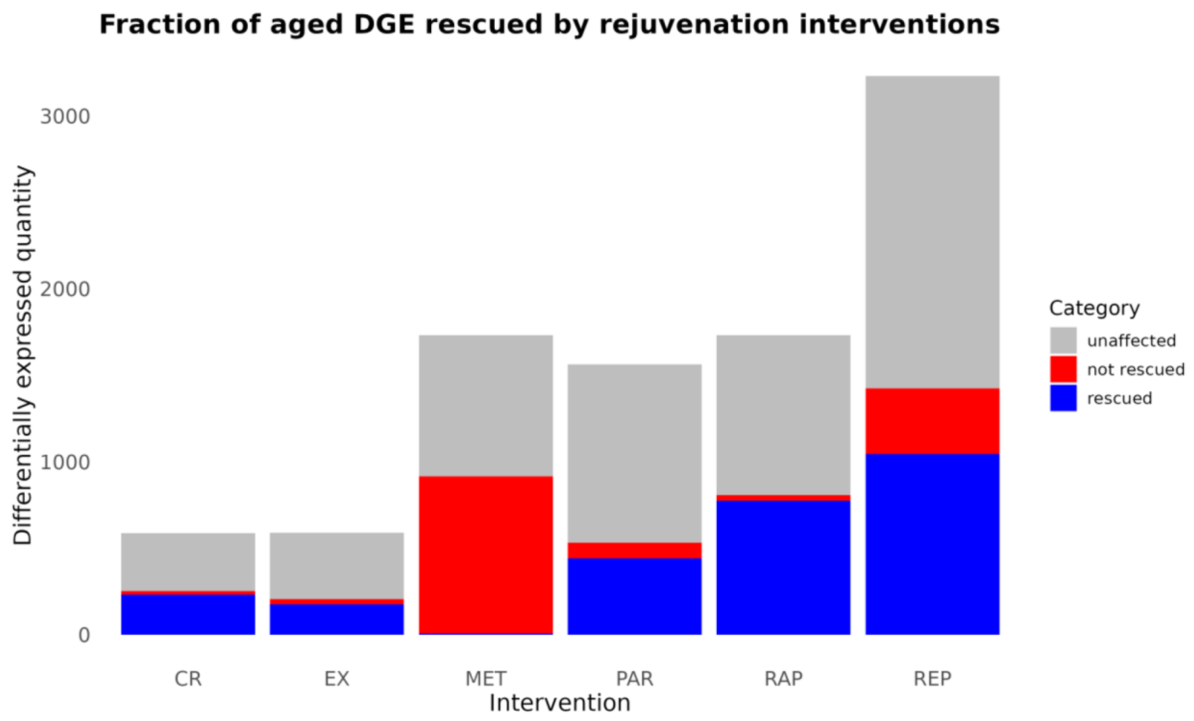

Supplementary Figure 1. Each bar represents, for a given intervention, the average size of the old versus young differential gene expression set across organs and cell types. This is further categorized into two types of genes in the intersection between genes seen in old versus young and rejuvenated versus old untreated: those where the sign of the change is different ('rescued') and those where the sign remains the same ('not rescued'). Given the sum of the rescued and not rescued fraction entails the genes for which we have information to compare, it is remarkable that with some heterogeneity, a very big fraction of the genes is rescued, meaning their expression state is successfully changed toward the state seen in young individuals. The noticeable exception is Metformin. A bbreviations: CR: Calorie Restriction; Ex: Exercise; Met: Metformin; Par: Parabiosis2; Rap: Rapamycin; Rep: Reprogramming2.

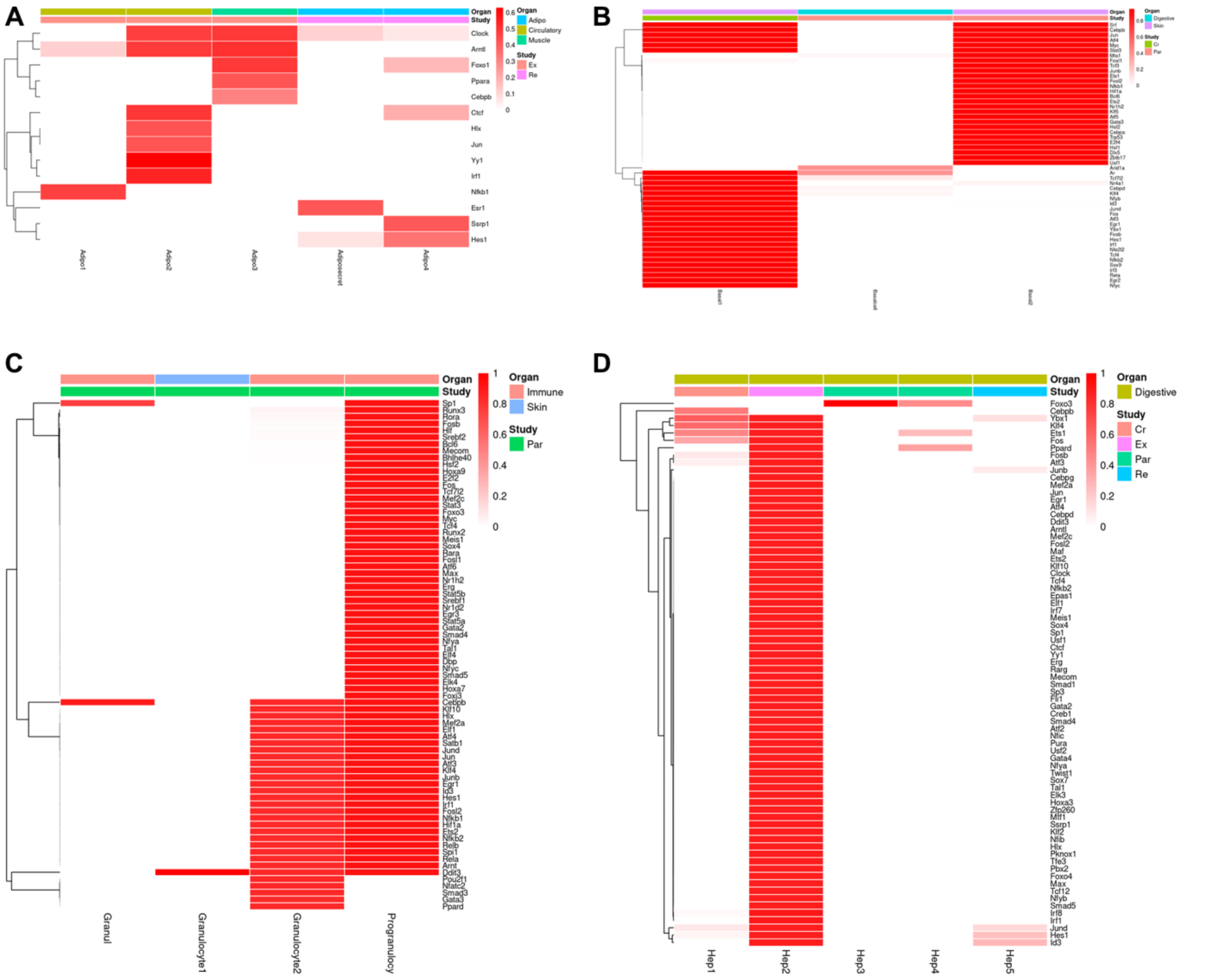

Supplementary Figure 2. (**A**) Heatmap of different TF scores for the Adipocyte celltype group. A shared Clock-Arntl mediated response can be seen twice the Exercise condition, but the downstream factors of this regulation change. (Values with no row with a value greater than .30 omitted). (**B**) Example of a highly dense network for two Basal cell types of the Skin. Srf, Cepbp Jun and Atf4 act in coordination in both cases, but the regulatory pathway downstream of that is almost entirely non-overlapping, and involves several transcription factors regulating each other. (Rows with no value greater than .30 omitted). (**C**) Different and shared rejuvenation factors, as well as less dense networks, after maturation of Granulocytes in the Parabiosis condition. (Rows with no value greater than .30 omitted). (**D**) Shared and distinct master regulators across and between interventions in Hepatocytes. (Rows with no value greater than .50 omitted). While Ybx1, Klf4, Ets1and Fos share a role in the rejuvenation response in both the Exercise and the Calorie Restriction conditions, two different datasets replicate involvement of Foxo3 instead in Parabiosis. This is an example of two candidate non-overlapping targets that could be investigated for additive or even synergistic rejuvenation benefits.

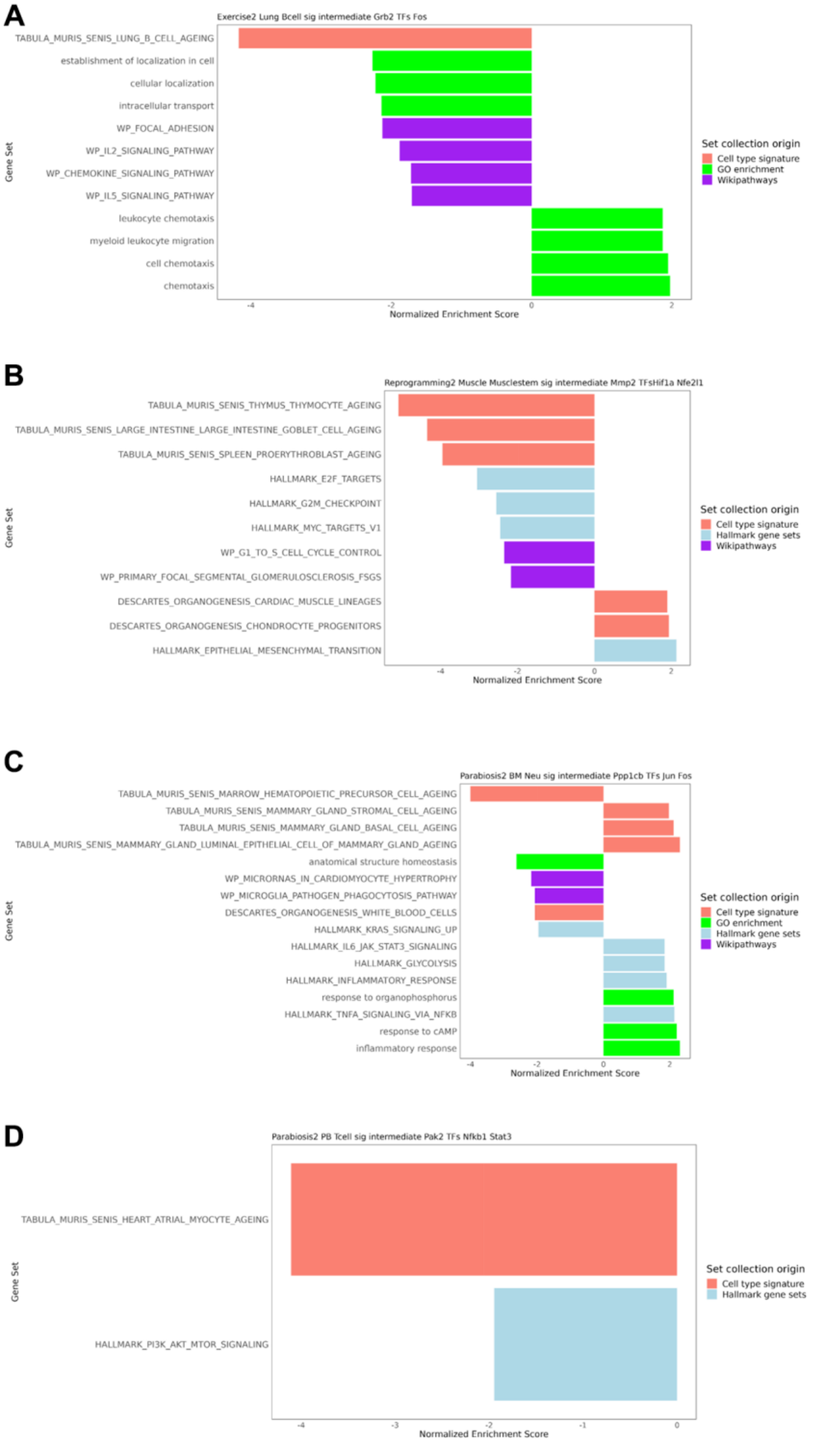

Supplementary Figure 3. Gene set enrichment analysis of genes belonging to selected signaling cascades in (**A**) Lung B cells after exercise, (**B**) Muscle stem cells after partial reprogramming, (**C**) Bone marrow neutrophils after parabiosis and (**D**) Peripheral blood T cells after parabiosis.
