# Supplementary material for: The cell rejuvenation atlas: leveraging network biology to identify master regulators of rejuvenation strategies
Source: Aging (Albany NY). 2024 Sep 9;16(17):12168–90. doi: 10.18632/aging.206105 (PMC11424581; doi:10.18632/aging.206105)
Supplement: Supplementary Tables [file aging-16-206105-s002.pdf]

SUPPLEMENTARY TABLES

Supplementary Table 1. GEO and GSA accession numbers of datasets used in this study.

Dataset	Accession number
Calorie Restriction	GSE137869
Exercise	CRA007207
Metformin	GSE194386
Parabiosis1	GSE193093
Parabiosis2	CRA004660
Parabiosis3	GSE222510
Rapamycin/Metformin	GSE210669
Reprogramming	GSE144600
Reprogramming2	GSE176206

Supplementary Table 2. Mapping of gene set names (column "Long name") to their corresponding abbreviation (column "Short name").

Long name	Short name
TABULA_MURIS_SENIS_LUNG_B_CELL_AGEING	Bcell aging
TABULA_MURIS_SENIS_HEART_AND_AORTA_FIBROBLAST_OF_CARDIAC_TISSUE_AGEING	Cardiac aging
TABULA_MURIS_SENIS_SPLEEN_PROERYTHROBLAST_AGEING	Proerythroblast aging
TABULA_MURIS_SENIS_MARROW_PRECURSOR_B_CELL_AGEING	Hsc Bell aging
TABULA_MURIS_SENIS_KIDNEY_KIDNEY_LOOP_OF_HENLE_THICK_ASCENDING_LIMB_EPITHELIAL_CELL_AGEING	Kidney aging
TABULA_MURIS_SENIS_MARROW_HEMATOPOIETIC_PRECURSOR_CELL_AGEING	Bm precursor aging
TABULA_MURIS_SENIS_MAMMARY_GLAND_STROMAL_CELL_AGEING	Stromal aging
TABULA_MURIS_SENIS_MAMMARY_GLAND_BASAL_CELL_AGEING	Basal cell aging
TABULA_MURIS_SENIS_MAMMARY_GLAND_LUMINAL_EPITHELIAL_CELL_OF_MAMMARY_GLAND_AGEING	Epithelial aging
TABULA_MURIS_SENIS_BRAIN_NON_MYELOID_NEURON_AGEING	Neuron aging
TABULA_MURIS_SENIS_GONADAL_ADIPOSE_TISSUE_MYELOID_CELL_AGEING	Myeloid aging
TABULA_MURIS_SENIS_MAMMARY_GLAND_T_CELL_AGEING	Tcell aging
TABULA_MURIS_SENIS_LIMB_MUSCLE_MESENCHYMAL_STEM_CELL_AGEING	Mesenchymal aging
TABULA_MURIS_SENIS_SKIN_BULGE_KERATINOCYTE_AGEING	Skin aging
TABULA_MURIS_SENIS_BRAIN_MYELOID_MACROPHAGE_AGEING	Macrophage aging
TABULA_MURIS_SENIS_SPLEEN_MATURE_NK_T_CELL_AGEING	Nkcell aging
TABULA_MURIS_SENIS_MARROW_NAIVE_B_CELL_AGEING	Bm Bcell aging
TABULA_MURIS_SENIS_LUNG_NON_CLASSICAL_MONOCYTE_AGEING	Monocyte aging
TABULA_MURIS_SENIS_GONADAL_ADIPOSE_TISSUE_MESENCHYMAL_STEM_CELL_OF_ADIPOSE_AGEING	AdiMes aging
TABULA_MURIS_SENIS_SPLEEN_T_CELL_AGEING	SpleenTc aging
TABULA_MURIS_SENIS_HEART_ATRIAL_MYOCYTE_AGEING	Myocyte aging
TABULA_MURIS_SENIS_THYMUS_THYMOCYTE_AGEING	Thymus Tc Aaging

TABULA_MURIS_SENIS_LARGE_INTESTINE_LARGE_INTESTINE_GOBLET_CELL_AGEING	Goblet aging
TABULA_MURIS_SENIS_SPLEEN_PROERYTHROBLAST_AGEING	Eryth Spleen aging
TABULA_MURIS_SENIS_LUNG_B_CELL_AGEING	Lung Bc aging
TABULA_MURIS_SENIS_LUNG_B_CELL_AGEING	LungBc aging
TABULA_MURIS_SENIS_SPLEEN_MACROPHAGE_AGEING	Spleen Mcr Aging
TABULA_MURIS_SENIS_SPLEEN_GRANULOCYTE_AGEING	Spleen Gran Aging
TABULA_MURIS_SENIS_LUNG_INTERMEDIATE_MONOCYTE_AGEING	Lung Mon Aging
TABULA_MURIS_SENIS_SPLEEN_B_CELL_AGEING	Spleen Bc Aging

Supplementary Table 3. Average age predictions from MultiTimer after mock treatment (column "Control_Avg_Age") or TF inhibition or upregulation (column "Experimental_Avg_Age"). Positive and negative age differences (Age_Difference) correspond to a pro- and anti-aging effect, respectively.

GSE Series	CellType	Method	Gene	Control_Avg_Age	Experimental_Avg_Age	Age_Difference
GSE114284	BEAS-2B	ko	IRF1 + IFNB	30.4647042486172	34.3877386364404	3.92303438782321
GSE140026	M0 Macrophages	ko	ATF4	29.1668560993142	27.4402233049184	−1.7266327943958
GSE140026	M1 Macrophages	ko	ATF4	59.5196662856039	57.7682136158112	−1.75145266979273
GSE140026	M2 Macrophages	ko	ATF4	50.9511918034666	48.4115306709847	−2.53966113248191
GSE140990	NHK	siRNA	KLF4	32.7457159077375	41.8536621191276	9.10794621139004
GSE56989	Primary human macrophages	siRNA	HIF1A	42.2828591457769	44.9064838639297	2.62362471815279
GSE73550	Endometrial stromal cells	siRNA	ESR1	29.9029503299127	27.8938114659652	−2.00913886394748
GSE90982	HUVECs	knockin/ upregulate	KLF4	33.6684708599789	35.3380624939053	1.66959163392637
GSE106502	MCF10A	knockin/ upregulate	MYC	41.4889946189905	31.7056828632328	−9.78331175575767

Supplementary Table 4. Gene set enrichment results for the signaling cascade including the intermediate Gnai2 and master regulators *Fos* and *Cebpb* in bone marrow macrophages after parabiosis treatment.

geneSet	Normalized EnrichmentScore	FDR	size	userId	Set collection origin
Spleen Proerythroblast Ageing	−1.89681927021782	0.0480488114910385	19	Atf4;Bsg;Cdk4;Cited2;Emd;Gapdh;Hmgb1;Hsp90aa1;Hspa5;Ldha;Pgk1;Prdx3;Rbbp7;Sdhb;Sod1;Stmn1;Top1;Tuba1b;U2af1	Cell type signature
Marrow Precursor B Cell Ageing	−1.83316975080196	0.0468624210838524	6	Akr1a1;Bsg;Fth1;Gapdh;Hmgb1;Sdhb	Cell type signature
nucleoside monophosphate metabolic process	−2.13758989447581	0.0084662592850568	5	Hif1a;Hspa8;Ldha;Pgk1;Stat3	GO enrichment
purine nucleoside monophosphate metabolic process	−2.13758989447581	0.0084662592850568	5	Hif1a;Hspa8;Ldha;Pgk1;Stat3	GO enrichment
nucleoside triphosphate metabolic process	−2.13758989447581	0.0084662592850568	5	Hif1a;Hspa8;Ldha;Pgk1;Stat3	GO enrichment

purine nucleoside triphosphate metabolic process	−2.13758989447581	0.0084662592850568	5	Hif1a;Hspa8;Ldha;Pgk1;Stat3	GO enrichment
ribonucleoside monophosphate metabolic process	−2.13758989447581	0.0084662592850568	5	Hif1a;Hspa8;Ldha;Pgk1;Stat3	GO enrichment
purine ribonucleoside monophosphate metabolic process	−2.13758989447581	0.0084662592850568	5	Hif1a;Hspa8;Ldha;Pgk1;Stat3	GO enrichment
ribonucleoside triphosphate metabolic process	−2.13758989447581	0.0084662592850568	5	Hif1a;Hspa8;Ldha;Pgk1;Stat3	GO enrichment
purine ribonucleoside triphosphate metabolic process	−2.13758989447581	0.0084662592850568	5	Hif1a;Hspa8;Ldha;Pgk1;Stat3	GO enrichment
ATP metabolic process	−2.13758989447581	0.0084662592850568	5	Hif1a;Hspa8;Ldha;Pgk1;Stat3	GO enrichment
HALLMARK_OXIDATIVE_PHOSPHORYLATION	−1.95707927282095	0.021953896816685	6	Atp6ap1;Cox17;Dlst;Ldha;Prdx3;Sdhb	Hallmark gene sets
WP_NUCLEAR_RECEPTORS	1.74347968154799	0.0457183805087782	5	Nr1d2;Nr4a1;Nr4a2;Rora	Wikipathways
regulation of chemotaxis	1.83367371500472	0.0328649544320262	9	Ccl4;Dusp1;Il1b;Vegfa	GO enrichment
regulation of leukocyte chemotaxis	1.8567723688262	0.0242731163447965	8	Ccl4;Dusp1;Il1b;Vegfa	GO enrichment
granulocyte migration	1.883399693367	0.0142310469350107	9	Ccl4;Cxcl2;Il1b;Vegfa	GO enrichment
granulocyte chemotaxis	1.883399693367	0.0142310469350107	9	Ccl4;Cxcl2;Il1b;Vegfa	GO enrichment
leukocyte migration	1.92899710894569	0.00678761916024705	11	Ccl4;Cxcl2;Dusp1;Il1b;Vegfa	GO enrichment
regulation of signaling receptor activity	1.95289017271554	0.00430374403276534	8	Ccl4;Cxcl2;Il1b;Osm;Vegfa	GO enrichment
taxis	1.971841009153	0.00247895656287284	16	Ccl4;Cxcl2;Dusp1;Il1b;Nr4a1;Nr4a3;Vegfa	GO enrichment
leukocyte chemotaxis	1.98973358621909	0.0025822464196592	10	Ccl4;Cxcl2;Dusp1;Il1b;Vegfa	GO enrichment
myeloid leukocyte migration	1.98973358621909	0.0025822464196592	10	Ccl4;Cxcl2;Dusp1;Il1b;Vegfa	GO enrichment
cell chemotaxis	1.99034656241804	0.00516449283931841	11	Ccl4;Cxcl2;Dusp1;Il1b;Nr4a1;Vegfa	GO enrichment
chemotaxis	2.00570057656868	0.00619739140718209	15	Ccl4;Cxcl2;Dusp1;Il1b;Nr4a1;Nr4a3;Vegfa	GO enrichment
